# Efficacy of glycogen synthase kinase-3β targeting against osteosarcoma via activation of β-catenin

**DOI:** 10.18632/oncotarget.12781

**Published:** 2016-10-20

**Authors:** Shingo Shimozaki, Norio Yamamoto, Takahiro Domoto, Hideji Nishida, Katsuhiro Hayashi, Hiroaki Kimura, Akihiko Takeuchi, Shinji Miwa, Kentaro Igarashi, Takashi Kato, Yu Aoki, Takashi Higuchi, Mayumi Hirose, Robert M Hoffman, Toshinari Minamoto, Hiroyuki Tsuchiya

**Affiliations:** ^1^ Department of Orthopedic Surgery, Graduate School of Medical Sciences, Kanazawa University, Kanazawa, Japan; ^2^ Department of Surgery, University of California, San Diego, CA, U.S.A; ^3^ AntiCancer Incorporated, San Diego, CA, U.S.A; ^4^ Division of Translational and Clinical Oncology, Cancer Research Institute, Kanazawa University, Kanazawa, Japan

**Keywords:** glycogen synthase kinase-3β, molecular target, treatment, osteosarcoma, orthotopic nude mice

## Abstract

Development of innovative more effective therapy is required for refractory osteosarcoma patients. We previously established that glycogen synthase kinase-3β (GSK- 3β) is a therapeutic target in various cancer types. In the present study, we explored the therapeutic efficacy of GSK-3β inhibition against osteosarcoma and the underlying molecular mechanisms in an orthotopic mouse model. Expression and phosphorylation of GSK-3β in osteosarcoma and normal osteoblast cell lines was examined, together with efficacy of GSK-3β inhibition on cell survival, proliferation and apoptosis and on the growth of orthotopically-transplanted human osteosarcoma in nude mice. We also investigated changes in expression, phosphorylation and co-transcriptional activity of β-catenin in osteosarcoma cells following GSK-3β inhibition. Expression of the active form of GSK- 3β (tyrosine 216-phosphorylated) was higher in osteosarcoma than osteoblast cells. Inhibition of GSK-3β activity by pharmacological inhibitors or of its expression by RNA interference suppressed proliferation of osteosarcoma cells and induced apoptosis. Treatment with GSK-3β-specific inhibitors attenuated the growth of orthotopic osteosaroma in mice. Inhibition of GSK-3β reduced phosphorylation at GSK- 3β-phospho-acceptor sites in β-catenin and increased β-catenin expression, nuclear localization and co-transcriptional activity. These results suggest the efficacy of GSK-3β inhibitors is associated with activation of β-catenin, a putative tumor suppressor in bone and soft tissue sarcoma and an important component of osteogenesis. Our study thereby demonstrates a critical role for GSK-3β in sustaining survival and proliferation of osteosarcoma cells, and identifies this kinase as a potential therapeutic target against osteosarcoma.

## INTRODUCTION

Osteosarcoma is the most common primary malignant bone tumor encountered during childhood and adolescence [1, 2]. Current treatment for this tumor is systemic chemotherapy using multiple agents in combination with limb-salvage surgery. The 5-year survival rate of patients with localized tumor treated by surgery alone was less than 20% prior to the introduction of chemotherapy, but has since been improved by chemotherapy to 70–90% [3, 4]. However, dose-intensive chemotherapy often leads to adverse effects and drug resistance, with some patients unable to tolerate the standard regimens. Despite more effective chemotherapy for primary tumors, the overall 5-year survival rate for patients with recurrent or metastatic osteosarcoma is just 20–30% and this has remained virtually unchanged for the past three decades [3]. Refractory patients should benefit from novel therapies that have less toxicity and resistance, including targeted therapies [3, 5].

Molecular-targeted therapies have been developed recently for the treatment of many cancer types, including osteosarcoma [3, 5, 6]. These targeted agents selectively affect molecules that are deregulated in tumor cells and are therefore associated with less toxicity to normal cells than chemotherapy involving cytotoxic drugs, such as cisplatinum and doxorubicin, which are currently first-line for osteosarcoma [3]. Some of the molecular-targeted agents evaluated in clinical trials for osteosarcoma are directed against receptor-type tyrosine kinases, intracellular signaling pathways, the bone microenvironment (eg., osteoclasts) and the immune system [5, 6]. Thus far, none have been approved for the treatment of osteosarcoma. The failure of some of these trials may be due to tumor heterogeneity [5] and to insufficient preclinical validation of the respective agents. Therefore, the identification of new molecular therapeutic targets has also been highlighted as a high priority for the treatment of refractory osteosarcoma [7, 8].

One emerging molecular target is glycogen synthase kinase-3β (GSK-3β), a serine/threonine protein kinase that maintains homeostasis in normal cells by regulating several fundamental biological pathways [9, 10]. Its known functions and involvement in various primary pathologies has established GSK-3β as a therapeutic target for type 2 diabetes mellitus, neurodegenerative diseases and inflammation [11]. In contrast to its effects against several proto-oncoproteins (eg., β-catenin, cyclin D1) and mediators of epithelial-mesenchymal transition in untransformed cells [12], we have found that deregulated GSK-3β facilitates the progression of gastrointestinal cancers and glioblastoma. This stems from the observation that GSK-3β promotes cell survival, proliferation and invasion by modulating distinct tumor suppressor pathways, cell immortality pathways and cell-motility machinery, thus rendering cancer cells resistant to chemotherapeutic agents and ionizing radiation [13–17]. Together with other studies, our observations have established GSK-3β as a potential therapeutic target in common adult-onset chronic diseases including cancer [13, 18, 19]. Therapeutic effects of GSK-3β inhibition have been shown in epithelial as well as in hematopoietic, neuronal and musculoskeletal cell malignancies including leukemia [20], glioblastoma [16, 17] and rhabdomyosarcoma [21, 22].

In the present study, we explored the expression, activity and potential pathological role of GSK-3β in osteosarcoma, a rare but devastating disease that predominantly affects children and young adults.

## RESULTS

### Expression and phosphorylation of GSK-3β

**Figure 1:**
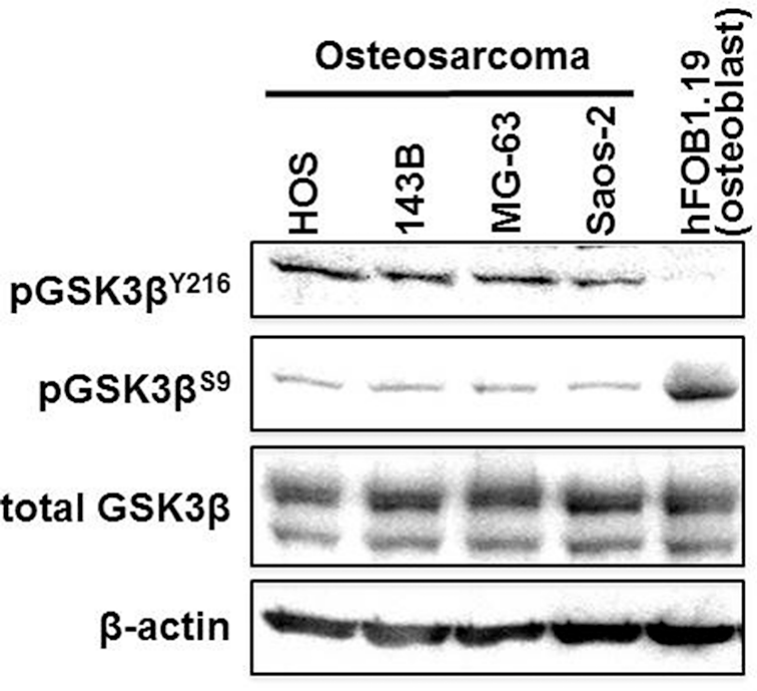
Expression and phosphorylation of GSK3β in osteosarcoma cells and in untransformed osteoblasts Fractions of phosphorylated GSK-3β (pGSK-3β^Y216^: active form; pGSK-3β^S9^: inactive form) and total GSK-3β were detected in protein extracts from osteosarcoma and hFOB1.19 cells by Western immunoblotting analysis. The amount of protein extract from each sample was compared to the expression of β-actin.

Osteosarcoma and osteoblast cells showed similar basal levels of GSK-3β expression (Figure 1). All osteosarcoma cell lines showed higher levels of pGSK- 3β^Y216^ (active form) and lower levels of pGSK- 3β^S9^ (inactive form) compared to hFOB1.19 osteoblast cells. These findings were consistent with our previous observations [14–16] and led us to hypothesize that osteosarcoma cells depend on deregulated GSK-3β for their survival and proliferation.

### Effects of GSK-3β inhibition on osteosarcoma cells

**Figure 2:**
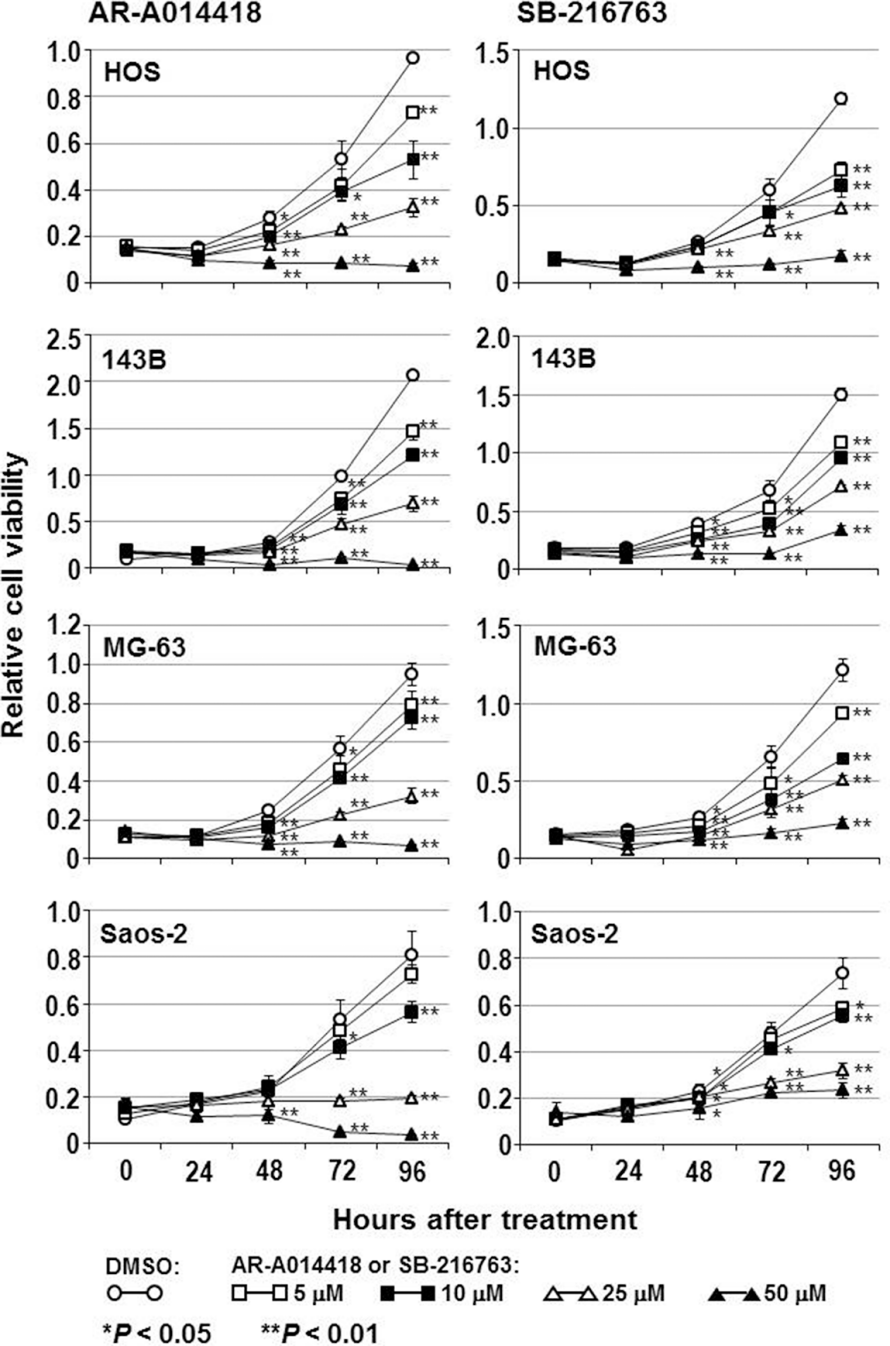
Effect of small-molecule GSK-3β inhibitors on the survival of osteosarcoma cells The osteosarcoma cells were treated with DMSO or the indicated concentrations of AR-A014418 or SB-216763 for the designated times. The relative number of viable cells at each time point was measured by the WST-8 assay. Values shown are the means ± SD of six separate experiments.

**Figure 3:**
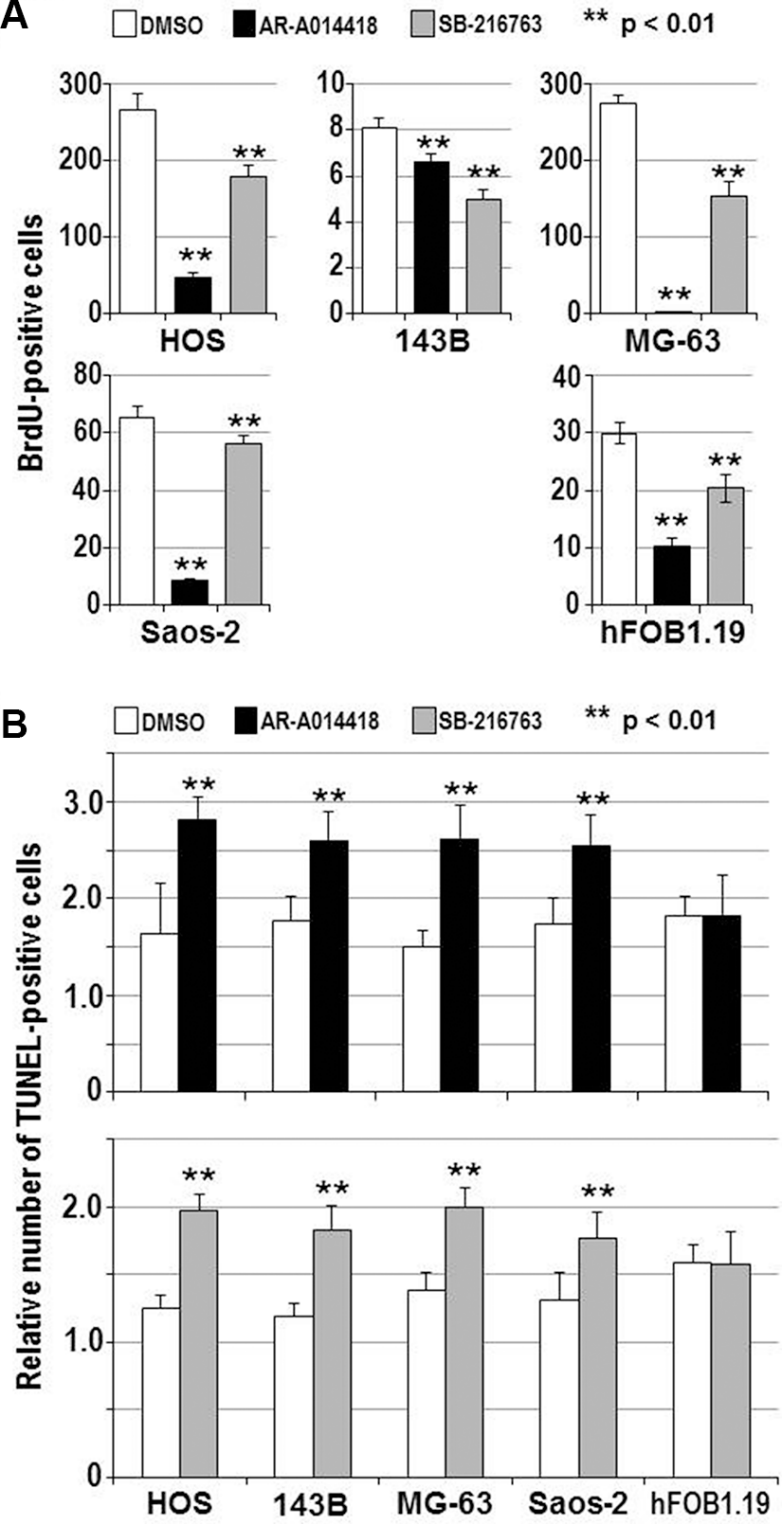
Effects of GSK-3β inhibitors on the proliferation and apoptosis of osteosarcoma cells and osteoblasts (**A**) The indicated cells were treated with DMSO or 25 μmol/L each of either AR-A014418 or SB-216763 for 72 hours. The relative number of proliferating cells was evaluated by measuring the amount of BrdU incorporation. (**B**) Relative numbers of TUNEL-positive apoptotic cells were scored for the indicated cells at 12 hours after treatment with DMSO or 25 μmol/L each of either GSK-3β inhibitor. (A, B) Values shown are the means ± SD of six separate experiments. Asterisks denote a statistically-significant difference (*p* < 0.01) between cells treated with DMSO and either GSK-3β inhibitor.

**Figure 4:**
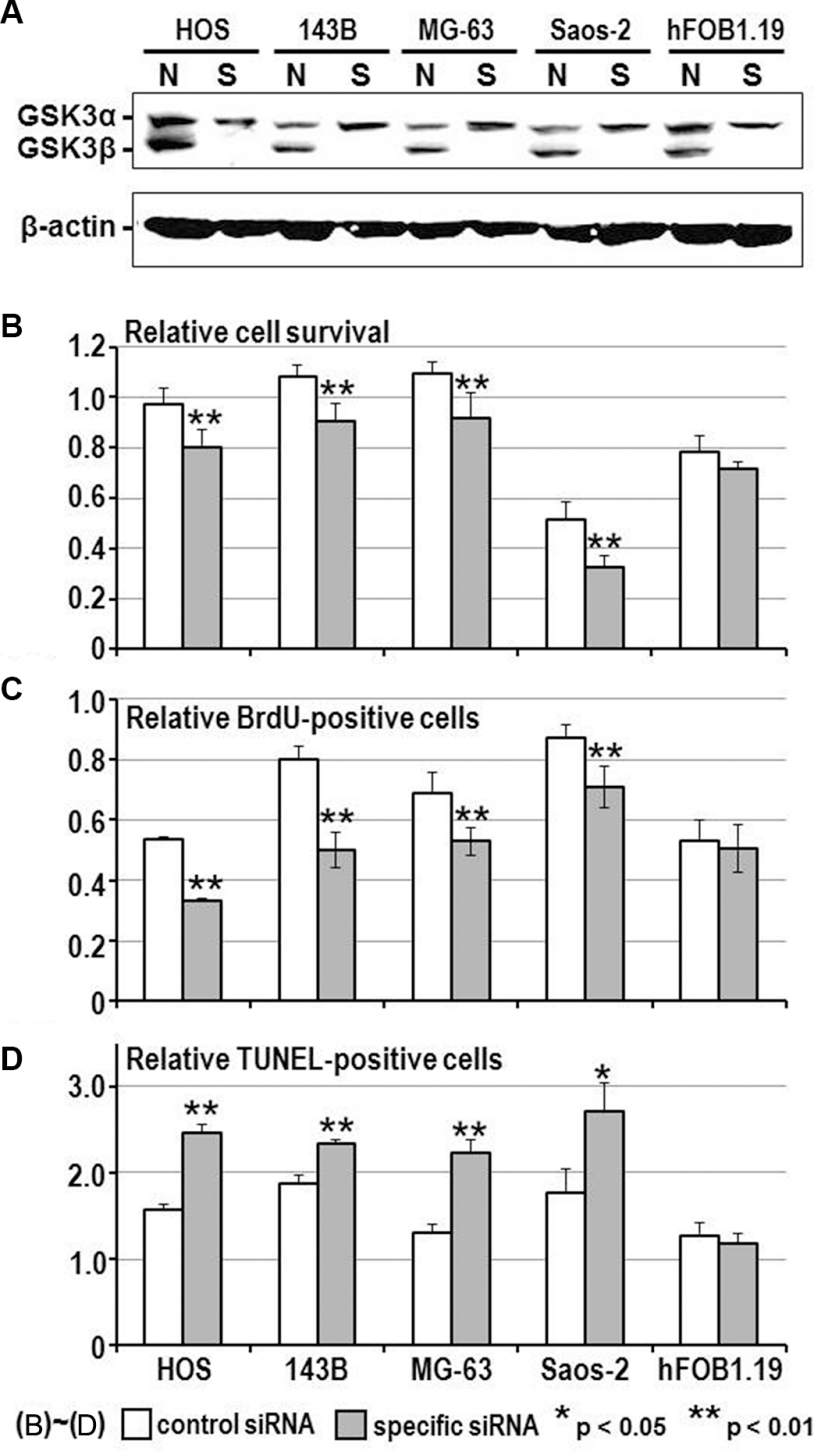
Effect of RNA interference on the expression of GSK-3β, cell viability, proliferation and apoptosis in osteosarcoma and osteoblast cells (**A**) Western-blotting analysis compared the level of expression of GSK-3α and GSK-3β between cells treated with non-specific (N) and GSK-3β-specific (S) siRNA (20 nmol/L each), respectively. Expression of β-actin was monitored as a loading control. (**B**–**D**) Relative number of surviving cells, BrdU-positive proliferating cells and TUNEL-positive apoptotic cells were counted and compared between cell types 96 hours after transfection of non-specific and GSK3β-specific siRNA. Values shown are the mean ± SD of six separate experiments. Asterisks denote a statistically-significant difference between cells transfected with non-specific and GSK- 3β-specific siRNA.

To address the above hypothesis, we examined the effect of GSK-3β inhibition on osteosarcoma cell survival and proliferation. The viability of all osteosarcoma cells was reduced in a dose- and time-dependent manner following treatment with AR-A014418 or SB-216763 (Figure 2), while a less marked effect of these inhibitors was observed in hFOB1.19 osteoblast cells (Supplementary Figure S2). The half-maximal inhibitory concentration (IC_50_) values at 96 hours after administration of AR-A014418 were 12.4 μmol/L for HOS; 11.7 μmol/L for 143B; 16.0 μmol/L for MG-63; and 14.6 μmol/L for Saos-2 cells. IC50 values for SB-216763 were 10.5 μmol/L for HOS; 17.1 μmol/L for 143B; 14.3 μmol/L for MG-63; and 21.9 μmol/L for Saos-2 cells. These concentrations are within the pharmacological dose ranges for these inhibitors [23, 24]. The GSK-3β inhibitors significantly decreased the relative number of BrdU-positive proliferating osteosarcoma and osteoblast cells (Figure 3A) and increased the relative number of TUNEL-positive cells undergoing apoptosis in the osteosarcoma cell lines, but not in hFOB1.19 osteoblast cells (Figure 3B). Reduction of the level of GSK-3β expression by RNA interference affected osteosarcoma, but not osteoblast proliferation, apoptosis and survival (Figure 4). Consistent with a previous study [25], these results indicate that both activity and expression of GSK-3β are necessary for osteosarcoma cell survival and proliferation, thereby suggesting a potential tumor-promoting role for this kinase in osteosarcomas.

### Changes in subcellular localization and activity of β-catenin following GSK-3β inhibition

**Figure 5:**
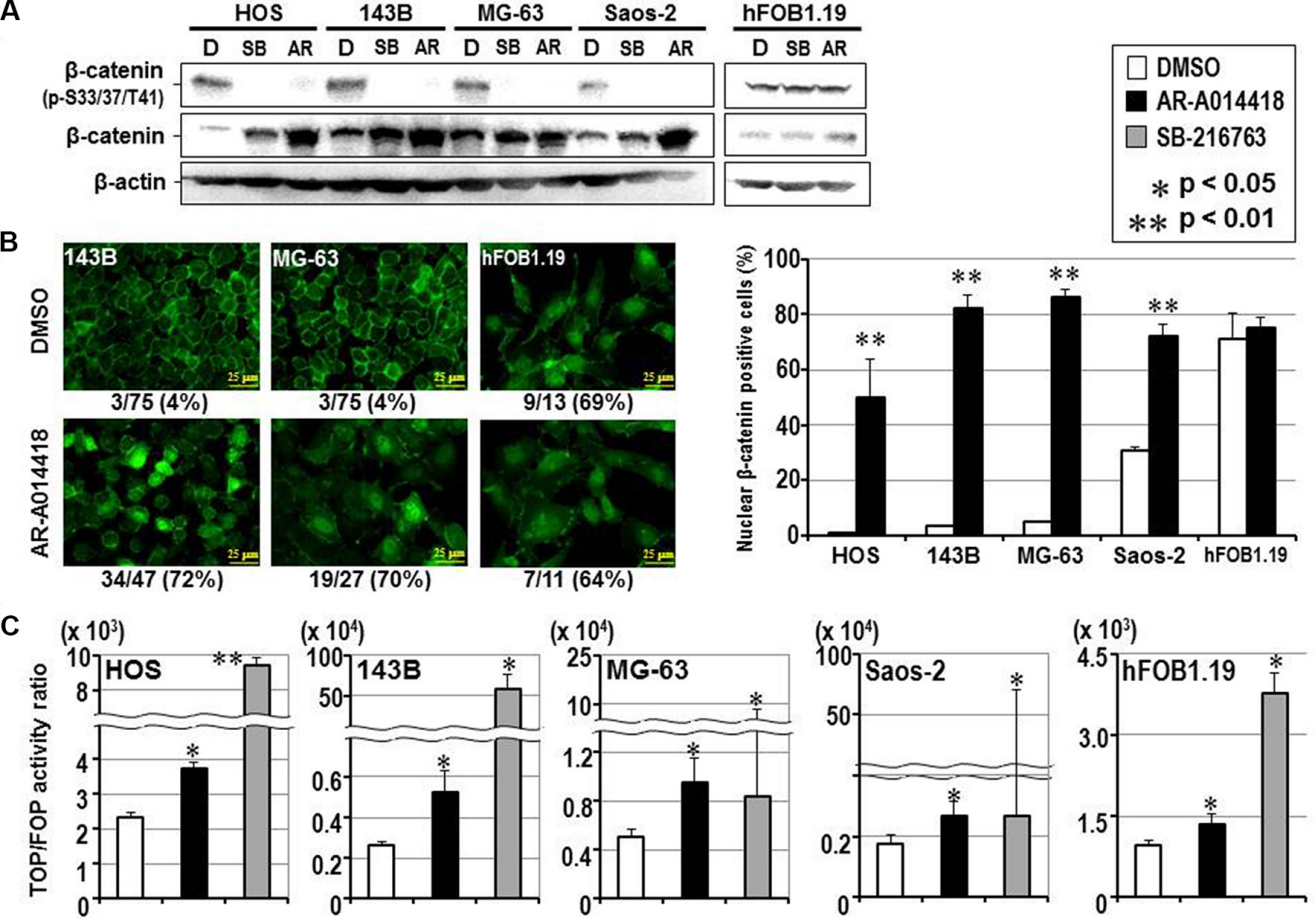
Effect of GSK-3β inhibition on the expression, phosphorylation, subcellular localization and co-transcriptional activity of β-catenin in osteosarcoma and osteoblast cells (**A**) Western-blotting analysis was used to compare the expression and phosphorylation of β-catenin between cells treated with DMSO and either GSK-3β inhibitor. Expression of β-actin was monitored as a loading control. (**B**) The left panels show representative immunofluorescence microscopic findings of expression and subcellular localization of β-catenin in osteosarcoma (143B, MG-63) and osteoblast (hFOB1.19) cells. The scale bar in each panel indicates 25 μm. The number shown below each panel indicates the percentage of nuclear β-catenin-positive cells among the total number of cells. The bar graphs on the right shows the effects of DMSO and AR-A014418 on the incidence of nuclear localization of β-catenin in osteosarcoma and osteoblast cells. In each assay, the mean percentage of nuclear β-catenin-positive cells in 3 microscopic fields was evaluated with standard deviation. (**C**) Relative co-transcriptional activity of β-catenin was measured by the TOP/FOP flash assay and compared between cells treated with DMSO, AR-A014418 and SB-216763, respectively. (B, C) Asterisks denote a statistically-significant difference between the data after administration of vehicle and GSK-3β inhibitors.

Previous studies showed the canonical Wnt/β-catenin pathway was inactivated during the development and progression of bone and soft tissue sarcomas including osteosarcoma [26–28]. In contrast to its oncogenic role in many cancer types [29, 30], this observation suggested a tumor suppressor function for the Wnt/β-catenin pathway in osteosarcoma [26–28]. Therefore, we focused on β-catenin, the downstream effector of the Wnt signaling pathway that is phosphorylated by GSK-3β for ubiquitin-mediated proteasomal degradation [29]. Consistent with the results shown above in Figure 1, β-catenin was phosphorylated at the known GSK-3β phospho-acceptor residues (S33, S37 and/or T41) in osteosarcoma cells. Treatment with GSK- 3β inhibitors suppressed the phosphorylation of β-catenin and increased its expression (Figure 5A). GSK-3β inhibitors also induced the nuclear translocation of β-catenin in osteosarcoma cells, whereas the nuclear localization of β-catenin was constitutively observed in hFOB1.19 osteoblasts regardless of treatment with DMSO or GSK-3β inhibitor (Figure 5B, Supplementary Figure S3). The TOP/FOP flash assay showed a significant increase in T-cell factor-dependent promoter activity in osteosarcoma cells following treatment with GSK-3β inhibitors (Figure 5C), reflecting an increase in the co-transcriptional activity of β-catenin.

The influence of β-catenin expression on the therapeutic effects of GSK-3β inhibition of osteosarcoma cells was examined by RNA interference of β-catenin prior to the treatment of cells with GSK-3β inhibitor. Depletion of β-catenin reduced the effects of AR-A014418 on the proliferation and apoptosis of Saos-2 to a greater extent than the other 3 osteosarcoma cell lines (143B, MG-63 and HOS) (Supplementary Figure S4). This suggests the therapeutic effect of GSK-3β inhibition depends not only on β-catenin induction and its co-transcriptional activity but also on known [13, 18] and/or unknown biological mechanism(s) that remain to be further investigated. Treatment with AR-A014418 attenuated proliferation of hFOB1.19 osteoblast cells under conditions of depleted β-catenin expression (Supplementary Figure S4), demonstrating a different role of β-catenin in normal osteoblasts and osteosarcoma cells.

It is of interest whether GSK-3β inhibition has therapeutic efficacy against osteosarcoma cells where increased β-catenin has been linked to malignancy. To address this issue, we investigated the activity of GSK- 3β inhibitors on survival of human osteosarcoma U2OS cells which were reported to depend on β-catenin signaling for cell proliferation [31]. Similar to other osteosarcoma cell lines (Figure 2), both GSK-3β inhibitors dose- and time-dependently suppressed U2OS cells survival (Supplementary Figure S5A). IC_50_ values at 96 hrs treatment for AR-A014418 and SB-216763 were 18.0 μmol/L and 33.7 μmol/L, respectively. The effect of GSK-3β inhibition was observed in the U2OS cells after knock-down of β-catenin expression (Supplementary Figure S5B), suggesting that β-catenin signaling-independent mechanism(s) such as alteration in NF-κB activity [25] may underlie the efficacy of GSK-3β inhibition against U2OS osteosarcoma cells.

### Efficacy of a GSK-3β inhibitor on AKT activity and NF-κB transcriptional activity

It is well documented that activation of phosphatidylinositol-3 kinase by its corresponding tyrosine kinase receptors results in the recruitment of AKT to the cytoplasmic membrane and its activation. AKT then functions upon multiple downstream effectors including GSK-3β [reviewed in 32]. Since AKT inactivates GSK- 3β by phosphorylation of its S9 residue, we examined whether GSK-3β regulates AKT activity in osteosarcoma and osteoblast cells. Western blotting analysis showed that the levels of AKT phosphorylation at its T308 and S473 residues (pAKT^T308^, pAKT^S473^), reflecting its activity [32], were higher in 3 of 4 osteosarcoma cell lines with low pGSK-3β^S9^ (as shown in Figure 1) than hFOB1.19 osteoblast cells (Supplementary Figure S6A). Treatment with 25 μmol/L AR-A014418 for 72 hrs did not change the levels of pAKT^T308^ and pAKT^S473^ in osteosarcoma and osteoblast cells. This is consistent with the fact that AKT functions upstream of GSK-3β [32] and with our previous study showing no correlation in the phosphorylation-dependent activities between AKT and GSK-3β in colorectal cancer [33].

Based on the pioneering study showing that GSK- 3β is required for NF-κB activation in mouse embryonic hepatocyte survival [34], previous studies focused on the role of GSK-3β in regulating NF-κB activity in cancer cells [25, 35, 36]. Consistent with these studies on pancreatic cancer and osteosarcoma [25, 35, 36], treatment with 25 μmol/L AR-A014418 for 72 hrs attenuated the relative transcriptional activity of NF-κB in 3 of 4 osteosarcoma cell lines, but not in hFOB1.19 osteoblast cells (Supplementary Figure S6B). Together with the previous study showing that GSK-3β inhibition suppressed NF-κB transcriptional activity in U2OS osteosarcoma cells [25], these results suggest that GSK-3β participates in osteosarcoma cell survival by activation of NF-κB and inactivation of the β-catenin osteosarcoma suppressor, in the context of cancer cell types with different genetic and molecular characteristics.

### Effect of GSK-3β inhibitors in an orthotopic mouse model of osteosarcoma

**Figure 6:**
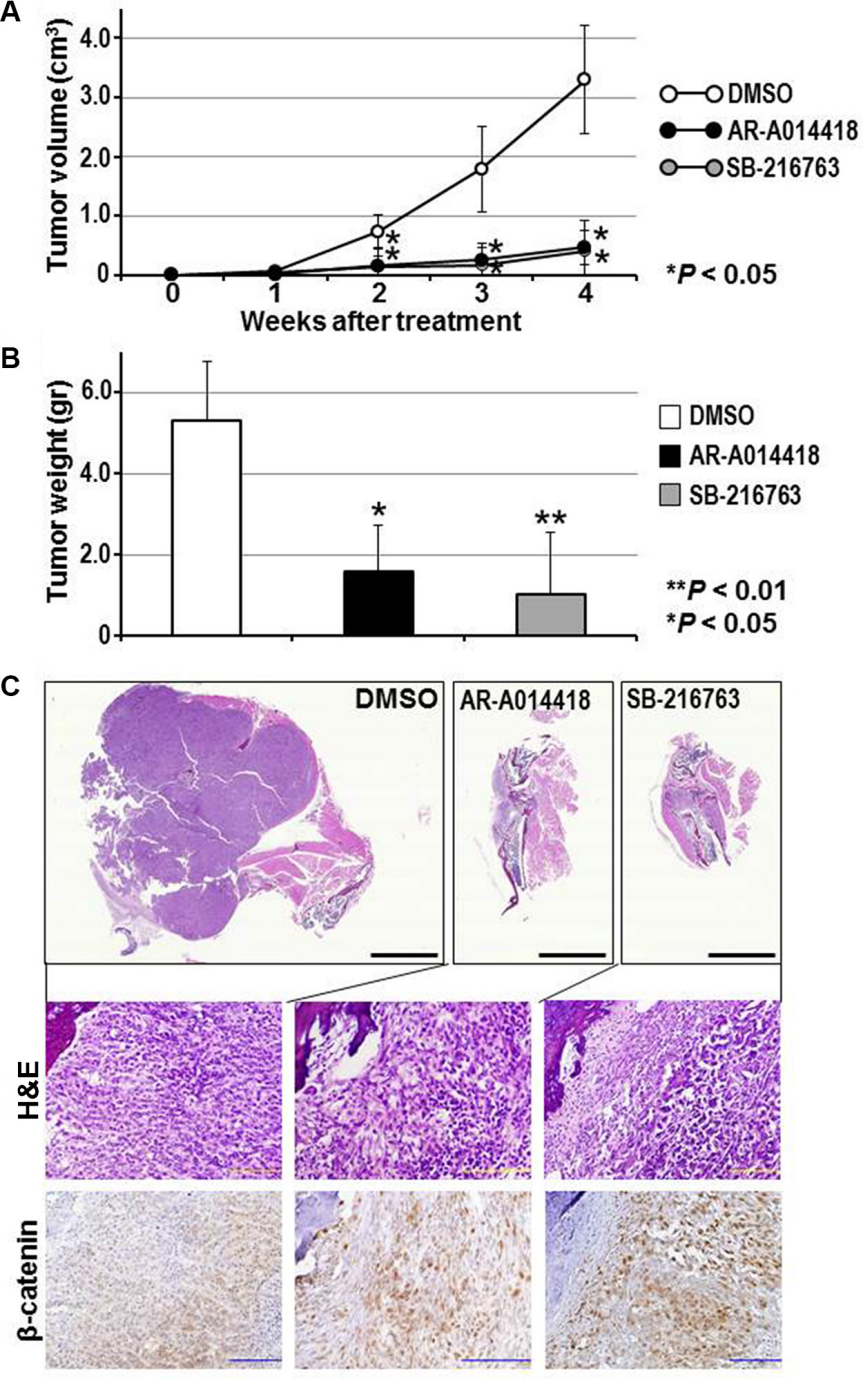
(**A, B**) Efficacy of GSK-3β inhibitors on the size and weight of orthotopic 143B tumors (A) Tumor size was measured weekly and the volume calculated. (B) Mean weight of the tumors removed at necropsy. Asterisks denote a statistically-significant difference in tumor volume and weight compared to mice treated with DMSO. The scatter plots corresponding to the data in (A) and (B) are shown in Supplementary Figure S7. (**C**) Histological and immunohistochemical findings for orthotopic tumors in mice treated with DMSO or with GSK-3β inhibitors. Representative paraffin-embedded sections of tumors were stained with H&E or immunostained for β-catenin. Magnified images of the sections (upper three panels) are shown in Supplementary Figure S8. Higher resolution versions of the lower six panels are shown in Supplementary Figure S9. Scale bar in each of the upper three panels indicates 5 mm and that of the middle and lower six panels indicates 100 μm.

Mice with orthotopic 143B tumors were treated by intraperitoneal injection of DMSO and either AR- A014418 or SB-216763 (2 mg/kg body weight each) three times per week for 5 weeks (Supplementary Figure S1). Administration of the GSK-3β inhibitors significantly reduced the tumor volume in mice as early as 2 weeks after treatment was initiated (Figure 6A, Supplementary Figure S7A). At the end of the study (8 weeks after treatment was started), tumors from GSK-3β inhibitor-treated mice weighed significantly less than those from the control group (Figure 6B, Supplementary Figure S7B). As reported in our previous studies [14, 15, 37, 38], no adverse effects were observed in mice during treatment with DMSO or either of the GSK-3β inhibitors. No significant differences in body weight were found between the groups of mice treated with DMSO, AR-A014418 and/or SB-216763. Gross observation and histological examination of the treated mice at autopsy revealed no pathological findings and no primary or metastatic tumors in the major vital organs including lungs, liver, gastrointestinal tract, pancreas and kidneys (data not shown).

Histological observation showed that orthotopically- transplanted cancer cells proliferated out from the tibia toward the surrounding limb tissue (Figure 6C, Supplementary Figure S8). The tumors in sham (DMSO)-treated mice showed medullary proliferation of tumor cells with plump cytoplasm. Compared to the controls, tumors in mice treated with GSK-3β inhibitors consisted of fibroblast-like cancer cells and were associated with reactive stromal and inflammatory cells. Immunohistochemically, nuclear localization of β-catenin in the cancer cells was frequently observed in tumors treated with GSK-3β inhibitors, whereas weak cytoplasmic expression was found in control tumors (Figure 6C; Supplementary Figure S9). The present experiment is proof-of-principle that GSK-3β inhibitors are active in a mouse models representing clinical osteosarcoma.

## DISCUSSION

Although several molecular targets with therapeutic potential have been proposed for osteosarcoma, the trials involving these targets have shown insufficient clinical benefit in refractory osteosarcoma patients [7, 8]. This has led us to address whether GSK-3β could be a clinically-useful target for the treatment of osteosarcoma. The present study found that osteosarcoma cells predominantly express the active form of GSK- 3β (pGSK-3β^Y216^) that we and others have shown contributes to cancer-cell survival, proliferation and invasion in several malignancies [14–17, 20–22]. Consistent with this observation, we demonstrate here that inhibition of either the activity or expression of GSK- 3β suppresses cell survival and proliferation and induces apoptosis in osteosarcoma cells. Furthermore, we have confirmed the efficacy of pharmacological GSK- 3β inhibitors against orthotopic tumor growth in mice. A large-scale experiment to further confirm these results will be performed in our future studies.

It is important to confirm the efficacy of the two inhibitors are specific to their effects against GSK-3β activity, and not non-specific or off-target effects. For this, we tested the GSK-3β-specific siRNA on osteosarcoma cells. In many cases, effect of an enzyme largely depends on its biological or catalytic activity rather than on its amount. GSK-3β is also the case and we previously reported that the pharmacological GSK-3β inhibitors (AR-A014418, SB-216763) inactivate GSK-3β in cancer cells within an hour after treatment and that the GSK-3β-specific siRNA takes longer than 48 hours to efficiently, but not completely, deplete GSK-3β expression in the same cells [38]. Therefore the pharmacological inhibitors more quickly inhibit GSK-3β activity in cells and lead to a stronger biological effect on cancer cells than treatment with GSK-3β-specific siRNA. However, both pharmacological inhibition of GSK-3β and depletion of GSK-3β by siRNA inhibited proliferation of osteosarcoma cells, suggesting that the pharmacological doses used to inhibit GSK-3β were on target. Together with an earlier study [25], our results confirm that osteosarcoma cells depend upon active GSK- 3β for their survival and proliferation, thus highlighting the potential benefit of targeting GSK-3β for the treatment of osteosarcoma.

An important novel finding relating to therapy was that GSK-3β inhibition had very little effect on the survival or apoptosis of untransformed hFOB1.19 osteoblasts. This was not reported in the earlier study [25] and is consistent with other studies showing that GSK-3β inhibition does not influence the survival or growth of non-neoplastic cells including human embryonic kidney cells (HEK293) [38] and embryonic lung and dermal fibroblasts (WI38, NIH- 3T3) [39]. Compared to osteosarcoma cells, we observed that hFOB1.19 osteoblast cells expressed a lower level of the active form (pGSK-3β^Y216^) and higher level of the inactive form (pGSK-3β^S9^) of this kinase. Our finding suggests that GSK-3β is inactive in normal cells, as we reported previously [15, 38, 33], thus rendering them less sensitive to GSK-3β inhibitors. The small effect of GSK-3β inhibitors on hFOB1.19 cells and large effect on mice with orthotopic tumors further support the use of these agents for treatment of osteosarcoma in the clinical setting. Larger scale experiments of the GSK-3β-targeted therapy will be performed in future studies.

An earlier study reported that GSK-3β promotes the survival and proliferation of osteosarcoma cells as well as their insensitivity to conventional chemotherapy [25], based on the known role for GSK-3β in positively regulating NF-κB-mediated signaling [35, 36]. Interestingly, in the current study we showed the therapeutic efficacy of GSK-3β inhibition was associated with increased expression, nuclear translocation and co-transcriptional activity of β-catenin. Although activation of the Wnt/β-catenin pathway has been associated with the development and progression of various cancer types such as colorectal cancer [29, 30], inactivation of this pathway has also been observed in bone and soft tissue sarcomas including osteosarcoma [26–28]. In contrast to an earlier study [25], the present results suggest that stabilization and activation of β-catenin may be a mechanism which underlies the efficacy of GSK-3β inhibition in osteosarcoma. To address this important issue, we need to identify molecular pathway(s) under the control of activated β-catenin following GSK-3β inhibition and this will be carried out in future experiments. Future experiments will also address osteosarcoma cells in which increased β-catenin is linked to malignancy. There is growing evidence the Wnt/β-catenin pathway plays a key role in bone formation and homeostasis by mediating osteoblastogenesis from progenitor cells, osteoblast differentiation, and by interfering with osteoclastogenesis [40–43]. It has been reported that osteoclasts in the tumor microenvironment facilitate the progression of osteosarcoma and hence these cells are considered a therapeutic target for osteosarcoma treatment [5, 6]. Thus, inhibition of GSK-3β in osteosarcoma may potentially confer three therapeutic advantages: activation of the β-catenin-mediated pathway to inhibit tumor progression; attenuation of the activity of tumor-associated osteoclasts; and the enhancement of local bone preservation.

The development and indication of GSK-3β inhibitors for the treatment of chronic diseases such as cancer requires a strong awareness of safety issues. The most serious concern is the possibility that long-term GSK-3β inhibition could initiate carcinogenesis due to the well-known role of this kinase in negatively regulating various proto-oncoproteins, in particular β-catenin [9–12, 29, 30]. To date, however, no studies have shown cellular neoplastic transformation via the activation of proto-oncoproteins, or demonstrated an increased incidence of cancer development or mortality following treatment with lithium, the classical GSK-3β inhibitor prescribed in clinical practice [44]. Primary tumor development was not observed in rodents following treatment with GSK-3β inhibitors in the present study or indeed in any previous studies [14, 15, 37, 38]. In contrast to its therapeutic efficacy against tumor-initiating (cancer stem) cells [45], inhibition of GSK-3β positively regulates the pluripotency of embryonic stem cells and the repopulation of hematopoietic stem cells via activation of the Wnt and hedgehog pathways, respectively [46, 47]. Moreover, GSK-3β inhibition has been shown to maintain the stemness of mesenchymal stem cells [48]. Therefore, inhibition of GSK-3β may provide indirect benefits for the treatment of osteosarcoma by limiting the extent of chemotherapy-associated bone marrow injury and by preserving tumor-adjacent stromal cells.

Despite recent advances in intensive multi-agent chemotherapy, tumor recurrence has been observed in 30–40% of osteosarcoma patients [49]. Tumor resistance to chemotherapeutic agents and a lack of suitable biomarkers for predicting the response to chemotherapeutic agents represent major obstacles for the improved treatment of refractory osteosarcoma patients [3, 5, 6]. A recent study showed that expression levels of GSK-3β, integrin β5 and focal-adhesion kinase in the primary tumors of osteosarcoma patients were significant predictors of poor response to neoadjuvant chemotherapy [50]. We previously reported that GSK-3β inhibition sensitizes pancreatic cancer and glioblastoma cells to gemcitabine and temozolomide, respectively, as well as sensitizing them to ionizing radiation through the modulation of distinct molecular pathways [14–17]. It is therefore important to address in future work whether GSK-3β inhibition can also modulate other chemotherapy drugs in osteosarcoma.

Previously-developed concepts and strategies of highly selective tumor targeting can take advantage of molecular targeting of tumors, including tissue-selective therapy which focuses on unique differences between normal and tumor tissues [51–56].

## MATERIALS AND METHODS

### Ethics statement

All animal studies were conducted with an AntiCancer Institutional Animal Care and Use Committee (IACUC)-protocol specifically approved for this study and in accordance with the principals and procedures outlined in the National Institute of Health Guide for the Care and Use of Animals under Assurance Number A3873-1. In order to minimize any suffering of the animals, anesthesia and analgesics were used for all surgical experiments. Animals were anesthetized by intramuscular injection of a 0.02 ml solution of 20 mg/kg ketamine, 15.2 mg/kg xylazine, and 0.48 mg/kg acepromazine maleate. The response of animals during surgery was monitored to ensure adequate depth of anesthesia. Ibuprofen (7.5 mg/kg orally in drinking water every 24 hours for 7 days post-surgery) was used in order to provide analgesia post-operatively in the surgically-treated animals. The animals were observed on a daily basis and humanely sacrificed by CO_2_ inhalation when they met the following humane endpoint criteria: prostration, skin lesions, significant body weight loss, difficulty in breathing, epistaxis, rotational motion and body temperature drop. The use of animals was necessary to evaluate drugs against osteosarcoma *in vivo*. Animals were housed with no more than 5 per cage. Animals were housed in a barrier facility on a high efficiency particulate arrestance (HEPA)-filtered rack under standard conditions of 12-hour light/dark cycles. The animals were fed an autoclaved laboratory rodent diet.

### Cell lines and culture

Human osteosarcoma (HOS, 143B, MG-63, Saos-2) [57–67] and osteoblast (hFOB1.19) [67] cell lines were obtained from the American Type Culture Collection (Manassas, VA) and maintained in Dulbecco's Modified Eagle Medium (DMEM, Wako, Osaka, Japan) and DMEM/Ham's F12 (Wako), respectively, both supplemented with 10% fetal bovine serum (FBS). The human osteosarcoma U2OS cell line is from the Human Cancer Cell Line Bank at the Kanazawa University Cancer Research Institute. The cells were maintained in RPMI-1640 (Sigma-Aldrich, St. Louis, MO) containing 10% FBS. This cell line was reported to depend on β-catenin signaling for its proliferation and invasion [31].

### Western blotting

Cellular protein was extracted with cell lysis buffer (CelLytic-MT, Sigma-Aldrich). A 30 μg aliquot of protein extract was analyzed by Western immunoblotting for the proteins of interest as described previously [15, 16]. The amount of protein in each sample was normalized to the expression of β-actin. The following primary antibodies were used at the dilutions shown against the indicated proteins: both GSK-3 isoforms (GSK-3α and β) (1:1,000, Millipore, Billerica, MA); GSK-3β (1:1,000, BD Biosciences, Lexington, KY); GSK-3β fractions phosphorylated at the serine (S) 9 residue (pGSK-3β^S9)^, (1:1,000, Cell Signaling Technology, Beverly, MA); and at the tyrosine (Y) 216 residue (pGSK-3β^Y216^) (1:1,000, BD Biosciences); β-catenin (1:1,000; BD Biosciences); β-catenin phosphorylated at S33, S37 and/or threonine (T) 41 residues (p-β-catenin^S33/37/T41^) (Cell Signaling Technology); AKT (1:1,000) and its fractions phosphorylated at T308 residue (pAKT^T308^) (1:1,000) and at S473 residue (pAKT^S473^) (1:1,000) (Cell Signaling Technology); and β-actin (1:4,000, Ambion, Austin, TX).

### Efficacy of GSK-3β inhibitors on cell survival, proliferation, and apoptosis *in vitro*

Cells seeded in 96-well plates were treated with dimethyl sulfoxide (DMSO) (Sigma-Aldrich) or the GSK-3β-specific inhibitors AR-A014418 (Calbiochem, San Diego, CA) [23] or SB-216763 (Sigma-Aldrich) [24] dissolved in DMSO and medium at the indicated concentrations that are within the reported pharmacological dose ranges [23, 24]. At the designated time points (0, 24, 48, 72, 96 hours) the relative number of viable cells was determined using a WST-8 (4-[3-(4-iodophenyl)-2-(4-nitrophenyl)-2H-5-tetrazolio]-1,3-benzene disulfonate) assay kit (Cell Counting Kit-8, Wako). Cell proliferation was evaluated following treatment with DMSO or with either of the two GSK- 3β inhibitors (25 μmol/L each) for 72 hours by using the BrdU enzyme-linked immunosorbent assay (ELISA) kit (Fluoroskan Ascent FL^®^; Labsystems, Thermo Fisher Scientific, Waltham, MA). Apoptosis was measured using a Click-iT terminal deoxynucleotidyl transferase-mediated dUTP nick end labeling (TUNEL) Alexa Fluor Imaging Assay kit (Life Technologies, Carlsbad, CA) following treatment with DMSO or with either GSK-3β inhibitor for 12 hours. In the assays for cell survival, proliferation and apoptosis, the values are expressed as the mean ± standard deviation (SD) of the 6-well set for DMSO and each inhibitor.

The specific effects of these small molecule inhibitors against GSK-3β activity are described in previous studies [23, 24]. These studies reported that while the biochemical IC_50_ values of AR-A014418 and SB-216763 are 104 nmol/l and 34.3 nmol/l, respectively, their pharmacological efficacy, such as on tau protein phosphorylation (AR-A014418) and on glucose incorporation into glycogen and activation of glycogen synthase (SB-216763) was observed in the dose range of 1 μmol/l to 50 μmol/l. Importantly, it was reported that 10 μmol/l AR-A014418 does not inhibit the activity of 26 closely related kinases and is therefore considered highly specific for GSK-3β [23]. Given the disparity in doses for biochemical compared to pharmacologic inhibition of GSK-3β described above, caution should be applied in studying these molecules. Since no subsequent information has been available for the effect of AR-A014418 on activity of kinases other than those reported previously [23], the effects of GSK-3β RNA interference were examined on cells transfected with either non-specific or GSK-3β-specific small interfering (si)RNA as described below.

### RNA interference

To examine the efficacy of GSK-3β expression on cell survival, proliferation and apoptosis, we used siRNA specific to human GSK-3β (target sequence, 5′- GCUCCAGAUCAUGAGAAAGCUAGAU-3′; GSK- 3β Validated Stealth RNAi) and negative control siRNA (Stealth RNAi Negative Control Low GC duplex), both purchased from Invitrogen, Life Technologies. The specificity of GSK-3β-specific siRNA was confirmed in our previous studies [15, 16]. The effect of siRNA on GSK-3β expression was observed by Western blotting with an antibody that recognizes both GSK-3α and GSK-3β (Millipore) as described above. Cells were seeded in 96- well plates and transfected with 20 nmol/l of either GSK-3β-specific or negative control siRNA using Lipofectamine RNAiMAX (Invitrogen). At 96 hours after transfection of siRNA, the relative number of viable cells and the extent of apoptosis were determined as described above.

The efficacy of GSK-3β inhibitors on osteosarcoma cell proliferation and apoptosis was examined under conditions of depleted β-catenin. Osteosarcoma cells were transfected with 15 nmol/l non-specific (SignalSilence control siRNA, Cell Signaling Technology) or β-catenin-specific siRNA (SignalSilence β-catenin siRNA II, Cell Signaling Technology) for 18 hrs. The cells were then seeded in a 96-well plate and examined for the efficacy of 25 μmol/l AR-A014418 on cell proliferation and apoptosis as described above. Prior to this, the efficiency of β-catenin knockdown in the cells was confirmed by Western blotting with antibody to β-catenin (BD Biosciences) as described above.

### Immunofluorescence and fluorescence staining

Osteosarcoma and osteoblast cells, grown on cover slips, were treated with either DMSO or 25 μmol/l AR-A014418 for 24 hrs and then fixed with 4% paraformaldehyde and permeabilized with 0.1% Triton-X (Sigma-Aldrich). The fixed and permeabilized cells were incubated with mouse monoclonal antibody to β-catenin (BD Bioscience; diluted 1:200) at 4°C overnight and then with Alexa Flour^®^ 488-labeled anti-mouse IgG (Invitrogen; diluted 1:1,000) at room temperature for 40 min. After washing off excess antibody, cell nuclei were counterstained with Hoechst 33342 (Molecular Probes) for 20 min at room temperature. The cells were observed by fluorescence microscopy (Keyence) for the expression and subcellular localization of β-catenin. The effect of GSK-3β inhibition on the subcellular localization of β-catenin was quantified in cells treated with DMSO or with AR-A014418. In each assay, the mean percentage of cells that was positive for nuclear β-catenin localization was calculated with SDs in 3 separate microscopic fields.

### TOP/FOP flash assay

To examine changes in the co-transcriptional activity of β-catenin following treatment with the GSK-3β inhibitors, we employed the TOP/FOP reporter system using the dual luciferase kit (Dual-GloTM Luciferase Assay System, Promega, Madison, WI) according to the manufacturer's instruction. The vectors used by this system were M50 Super 8X TOPFlash and M50 Super 8X FOPFlash (Addgene, Cambridge, MA). Cells were cultured for 14 hours and then transfected with both vectors using the Lipofectamine^®^ 2000 transfection reagent (Life Technologies). At 15 hours after transfection, cells were treated with DMSO, 25 μmol/l AR-A014418 or SB-216763 for 12 hours. Luciferase activity in the cells was then measured using a Centro LB 960 Microplate Luminometer (Berthold Technologies, Germany) at 12 hours after treatment with DMSO or with one of the two GSK-3β inhibitors. The relative value for the co-transcriptional activity of β-catenin in the cells is expressed as the mean ± SD of four separate experiments for the respective conditions performed in duplicate.

### Nuclear factor (NF)-κB transcriptional activity assay

Changes in NF-κB transcriptional activity following GSK-3β inhibition were measured for osteosarcoma and osteoblast cell lines with a luciferase reporter assay in the presence of DMSO or 25 μmol/L AR-A014418, according to our previous study [16]. This assay used the pGL4.32[*luc2P*/NF-κB-RE/Hygro] vector, pRL *Renilla* luciferase control vector (pRL-SV40 Vector) and the Dual-Luciferase^®^ Reporter Assay System (Promega). In each assay, the luciferase activity of pGL4.32[*luc2P*/NF-κB -RE/Hygro] vector-transfected cells was normalized to the co-transfected pRL-SV40 vector. The relative NF-κB transcriptional activity was then compared between the same cells treated with DMSO and 25 μmol/l AR-A014418 for 72 hrs.

### Orthotopic mouse model of osteosarcoma and treatment

The efficacy of GSK-3β inhibition on tumor growth was examined on 143B cells [58–65] transplanted orthotopically in athymic nude mice (AntiCancer, San Diego, CA). Twelve mice, six to eight weeks old, were anesthetized by a ketamine mixture (10 μl ketamine HCl, 7.6 μl xylazine, 2.4 μl acepromazine maleate, and 10 μl of sterilized water) via subcutaneous injection. The left leg was sterilized with alcohol and a 5 mm midline skin incision was made below the knee joint to expose the tibial tuberosity. 143B cells (5 × 10^5^ cells/5 μl) were suspended in Matrigel (BD Bioscience) and injected into the medullary cavity of the left tibia with a 0.5 ml 28 G latex-free insulin syringe (TYCO Health Group LP, Mansfield, MA). The skin was closed with a 6–0 suture as described previously [65]. These mice were assigned to 3 groups (4 mice each) and given intraperitoneal injection of 75% DMSO or of either GSK-3β inhibitor at a dose of 2 mg/kg body weight for three times per week and for 5 weeks (Supplementary Figure S1) as described in our previous studies [37, 38]. Assuming that 60% of body weight is accounted by body fluid, a dose of 2 mg/kg body weight corresponds approximately to a concentration of 10 μmol/l in culture medium for both inhibitors [37]. Throughout the experiment, all mice were carefully observed daily for any adverse events and the tumors were measured for size in two dimensions every week. Tumor volume (cm^3^) was calculated using the formula: 0.5 × *a^2^* × *b*, where *a* is the smallest tumor diameter (cm) and *b* is the largest [68].

All mice were euthanized after the completion of treatment. At necropsy, tumor and vital organs were removed and fixed in 10% neutral-buffered formalin. Tumor weight (g) was measured before fixation. Tumor tissues were decalcified after fixation and embedded in paraffin for histopathologic and immunohistochemical examination. Paraffin sections of the respective tumors were stained with hematoxylin and eosin (H&E) and immunostained with rabbit monoclonal antibody to β-catenin (diluted 1:100; Cell Signaling Technology) according to our previous studies [37, 38].

All animal experiments were undertaken in accordance with the Guidelines for the Care and Use of Laboratory Animals under National Institutes of Health assurance number A3873-01 and the U.S. Public Health Service Policy on Humane Care and Use of Laboratory Animals [69] that also covers the national guidelines in Japan [70].

### Statistical analysis

Data were evaluated statistically using the Student's *t* test (cell survival, proliferation and apoptosis) and Mann-Whitney *U* test (TOP/FOP Flash assay, volume of orthograft tumors); with a *p* value of < 0.05 considered statistically significant.

## SUPPLEMENTARY MATERIALS FIGURES



## Abbreviations

BrdU: 5-bromo-2′-deoxyuridine
DMSO: dimethyl sulfoxide
ELISA: enzyme-linked immunosorbent assay
FBS: fetal bovine serum
GSK-3β: glycogen synthase kinase-3β
NF-κB: nuclear factor-κB
SD(s): standard deviation(s)
siRNA: short interfering RNA
TUNEL: terminal deoxynucleotidyl transferase-mediated dUTP nick end labeling.
