# Supplementary material for: Efficacy of glycogen synthase kinase-3β targeting against osteosarcoma via activation of β-catenin
Source: Oncotarget. 2016 Oct 20;7(47):77038–51. doi: 10.18632/oncotarget.12781 (PMC5363568; doi:10.18632/oncotarget.12781)
Supplement: Supplementary file 1 [file oncotarget-07-77038-s001.pdf]

Efficacy of glycogen synthase kinase-3β targeting against osteosarcoma via activation of β-catenin

Supplementary Materials

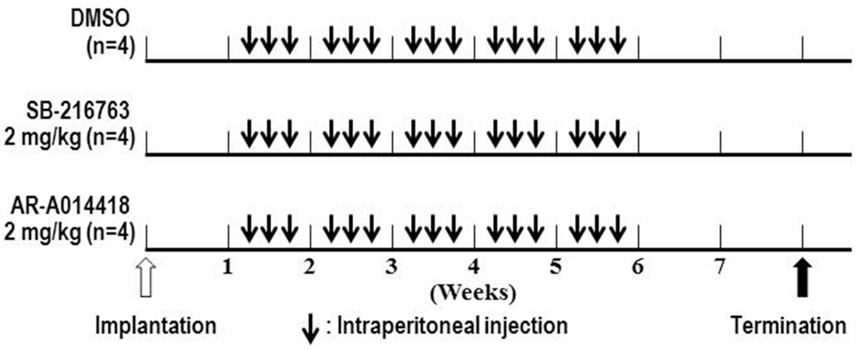

Supplementary Figure S1: Design and protocol of the animal experiment. At week 0, 143B cells (5×10^5) were inoculated into the medullary cavity of the left tibia of each mouse. The mice were randomly assigned to three groups for treatment with DMSO, SB-216763, or AR-A014418 by intraperitoneal injection three times per week. After 5 weeks of treatment, all mice were euthanized for necropsy.

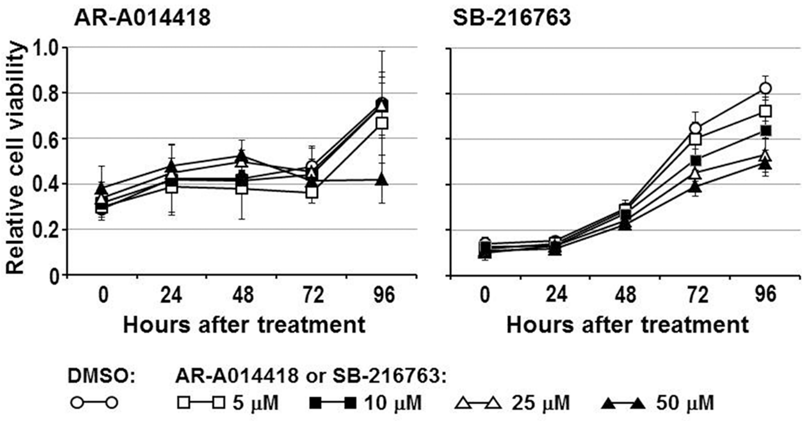

Supplementary Figure S2: Effect of small-molecule GSK3β inhibitors on the survival of osteoblast hFOB1.19 cells. The cells were treated with DMSO or the indicated concentration of AR- A014418 or SB-216763 for the designated times. The relative number of viable cells at each time point was measured by the WST-8 assay. Values shown are the means ± SD of six separate experiments.

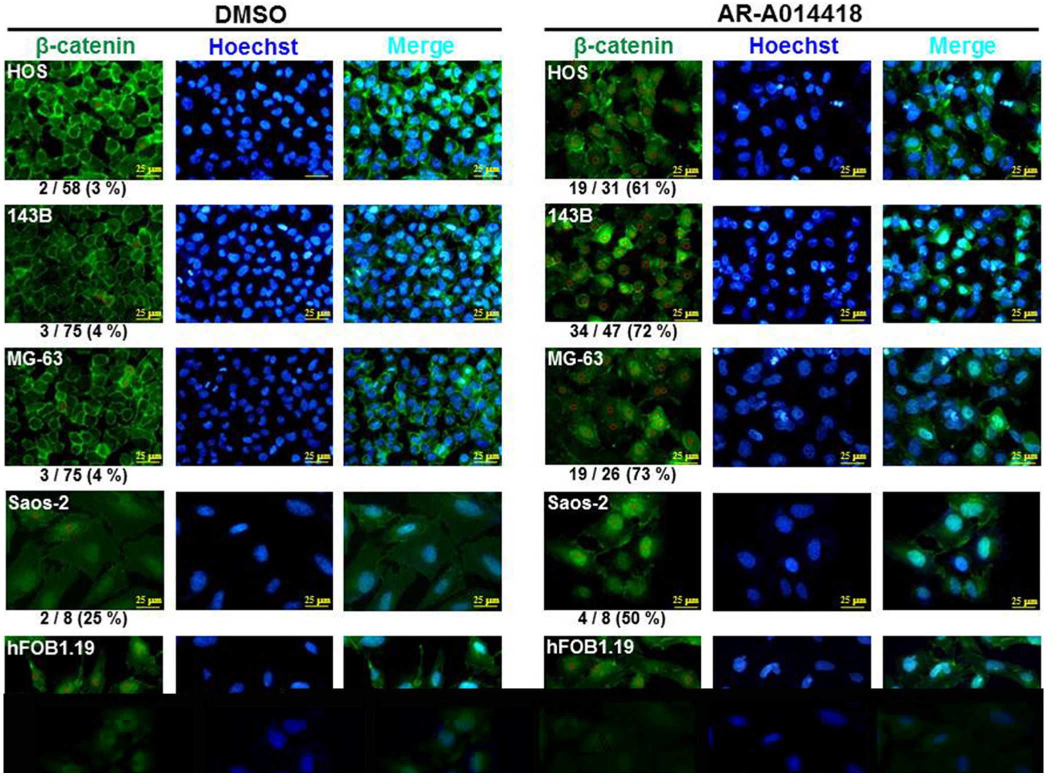

Supplementary Figure S3: Effect of GSK3β inhibition on expression and subcellular localization of β-catenin in osteosarcoma (HOS, 143B, MG-63, Saos-2) and osteoblast (hFOB1.19) cells. The cells were treated with 25 μmol/l AR-A014418 for 24 hrs and immunostained for β-catenin. Cell nuclei were counterstained with Hoechst 33342. Merged images are shown for the respective cells. The number shown below each panel indicates the percentage of nuclear β-catenin-positive cells among the total number of cells. The scale bar in each panel indicates 25 μm.

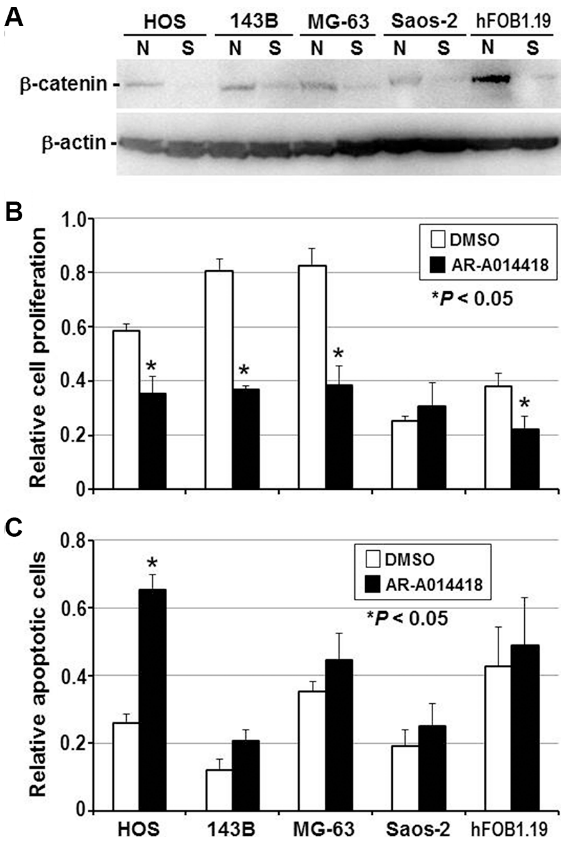

Supplementary Figure S4: Combined effects of GSK3β inhibition and depletion of β-catenin on the proliferation and apoptosis in osteosarcoma and osteoblast cells. (**A**) Osteosarcoma cells were transfected with non-specific (N) and β-catenin-specific (S) siRNA for 18 hrs. Protein extracts from them were then examined for β-catenin expression by Western blotting. Expression of β-actin was monitored as a loading control. (**B**, **C**) Effects of DMSO (D) and 25 μmol/l AR-A014418 (AR) on BrdU-positive proliferating cells (B) and TUNEL-positive apoptotic cells (C) were examined under depletion of β-catenin in osteosarcoma and osteoblast cells. Values shown are the means ± SD of three separate experiments.

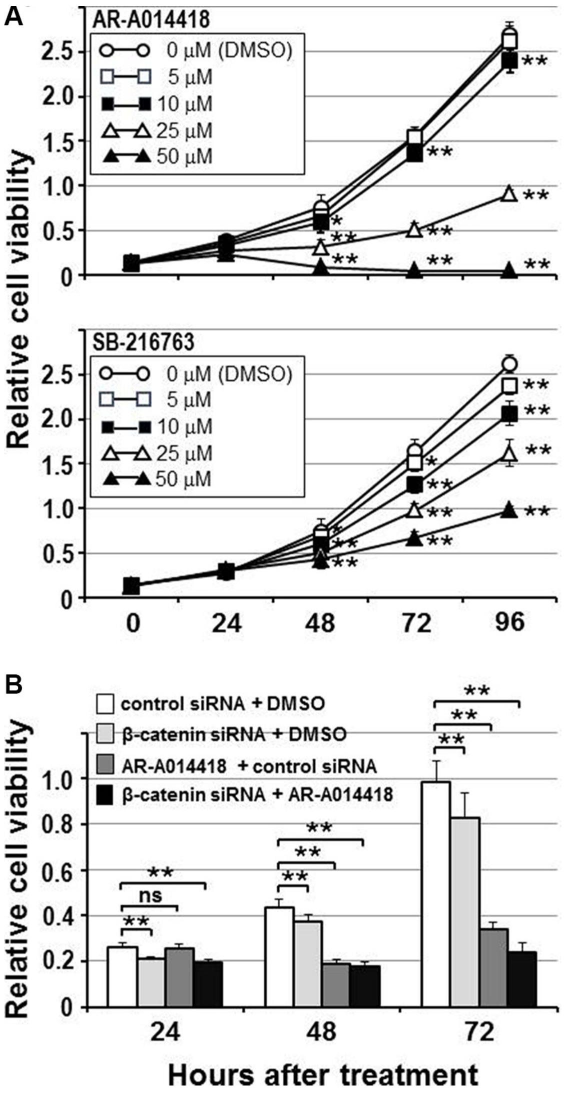

Supplementary Figure S5: Effect of small-molecule GSK3β inhibitors (A) and combined effect of GSK3β inhibitors and depletion of β-catenin (B) on the survival of U2OS osteosarcoma cells. (A) The cells were treated with DMSO or the indicated concentration of AR-A014418 or SB-216763 for the designated times. (B) The cells were transfected with non-specific (control) and β-catenin-specific siRNA, respectively, for 18 hrs. Effects of DMSO, β-catenin-siRNA and 25 µmol/l AR-A014418, alone or in combination, on cell survival were examined at the designated times following treatment. (A) and (B) The relative number of viable cells at each time point was measured by the WST-8 assay. Values shown are the means ± SD of six separate experiments. *$p < 0.05$; **$p < 0.01$; ns, no significant difference.

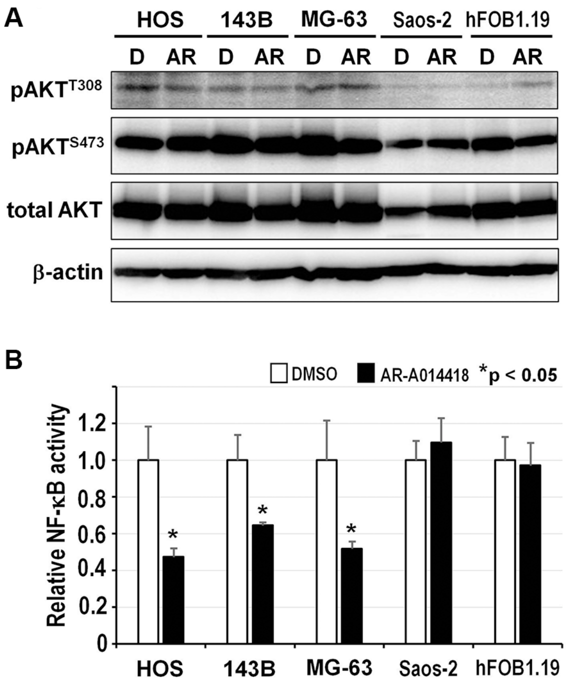

Supplementary Figure S6: Effects of GSK3β inhibition on the expression and phosphorylation of AKT (A) and on the transcriptional activity of NF-κB (B) in osteosarcoma and osteoblast cells. (A) The levels of expression and phosphorylation ($pAKT^{T308}$, $pAKT^{S473}$) of AKT were compared by Western blotting between the same cells treated with DMSO and 25 µmol/l AR-A014418, respectively, for 72 hrs. Expression of β-actin was monitored as a loading control. (B) Relative transcriptional activity of NF-κB was compared between the same cells treated with DMSO and 25 µmol/l AR-A014418, respectively, for 72 hrs by the luciferase reporter assay as described in the Methods. Values shown are the means ± SD of three separate experiments. *$p < 0.05$.

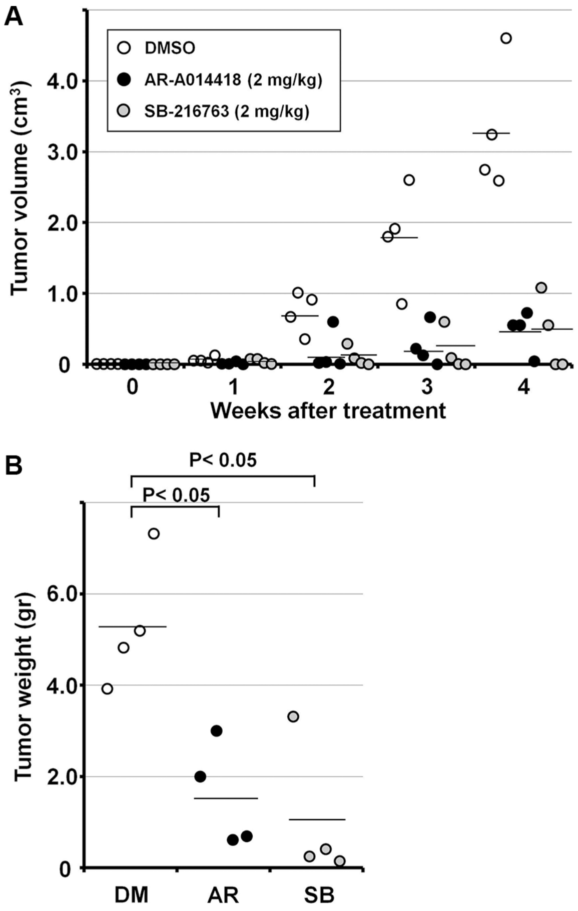

Supplementary Figure S7: Scatter plots of the data on the efficacy of GSK3β inhibitors on the size (volume) (A) and weight (B) of orthotopic 143B tumors shown in Figure 6A and 6B. The bar in each group of circles indicates a mean value. DM, DMSO; AR, AR-A014418; SB, SB-216763.

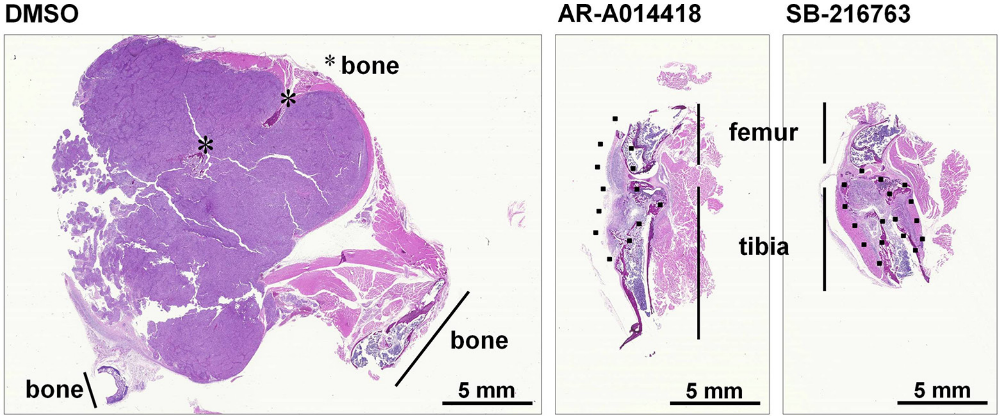

Supplementary Figure S8: Magnified images of the H&E-stained representative sections (Figure 6C) of the left lower limbs of mice where orthotopically inoculated 143B osteosarcoma cells proliferated. Dotted lines delineate the tumor lesion in the mice following treatment with 2 mg/kg each of AR-A014418 and SB-216763, three-time a week for 5 weeks (Figure S1).

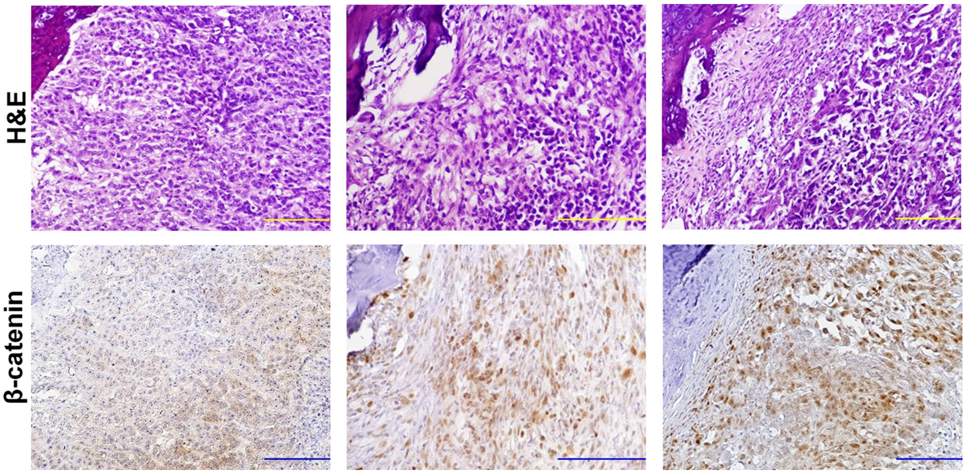

Supplementary Figure S9: Higher resolution version of the lower six panels in Figure 6 (C). Scale bar in each panel: 100 μm.
